# Supplementary material for: The GeoTox Package: Open-source software for connecting spatiotemporal exposure to individual and population-level risk
Source: medRxiv. 2024 Sep 24:2024.09.23.24314096. Preprint. [Version 1] doi: 10.1101/2024.09.23.24314096 (PMC11469396; doi:10.1101/2024.09.23.24314096)
Supplement: Supplementary file 1 [file media-1.pdf]

1 Supporting Information

This is the Supporting Information accompanying *The GeoTox Package: Open-source software for connecting spatiotemporal exposure to individual and population-level risk*. This document contains figures S1, S2, and S3.

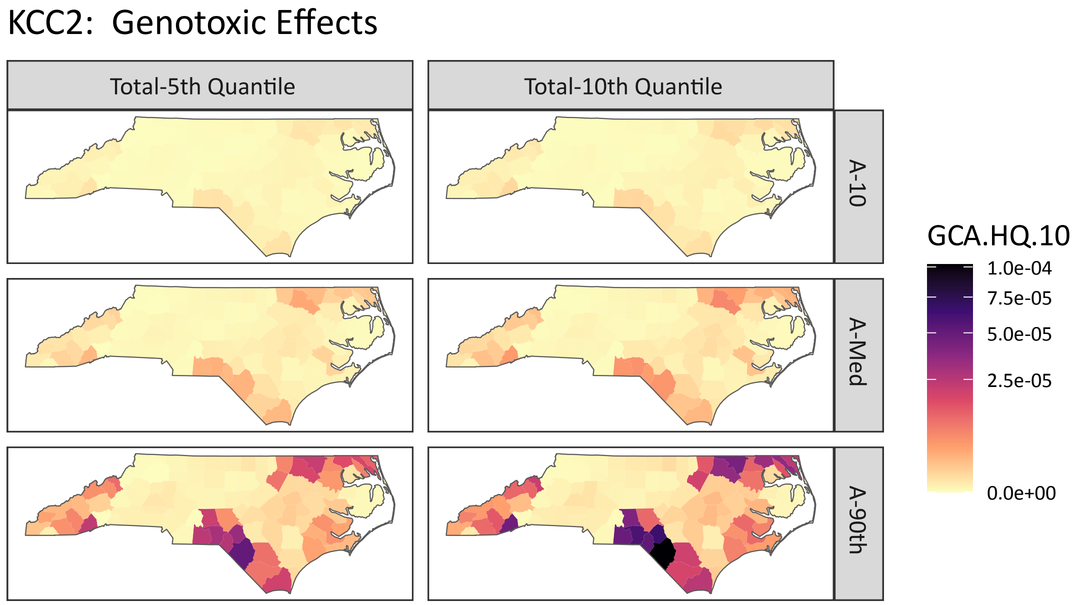

Figure S1: Multi-assay risk summaries for Key Characteristic of Carcinogens group 5 (genotoxic effects) mapped across North Carolina counties

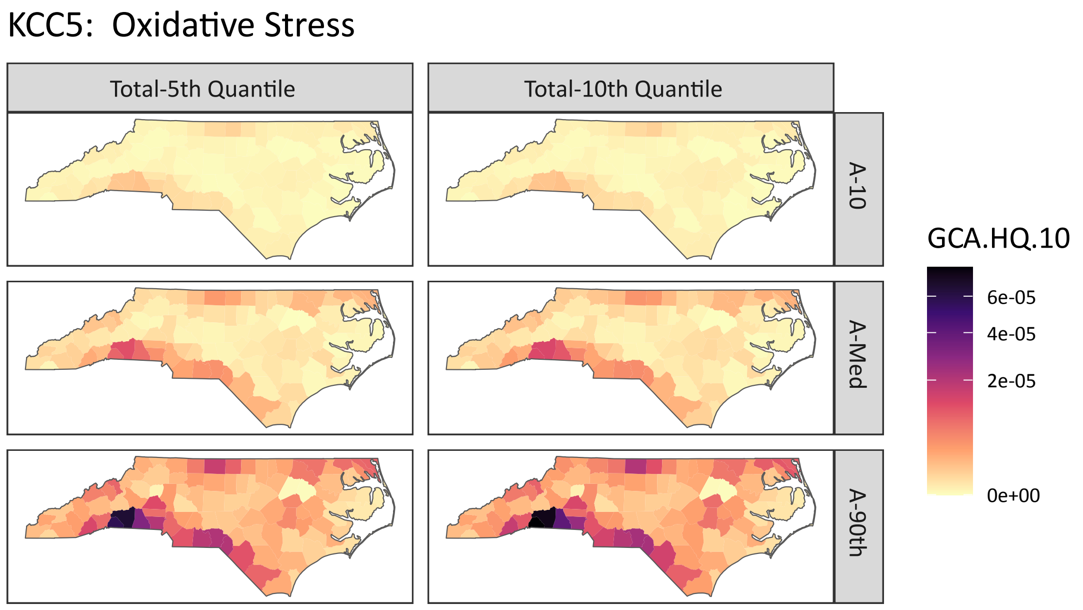

Figure S2: Multi-assay risk summaries for Key Characteristic of Carcinogens group 5 (oxidative stress) mapped across North Carolina counties

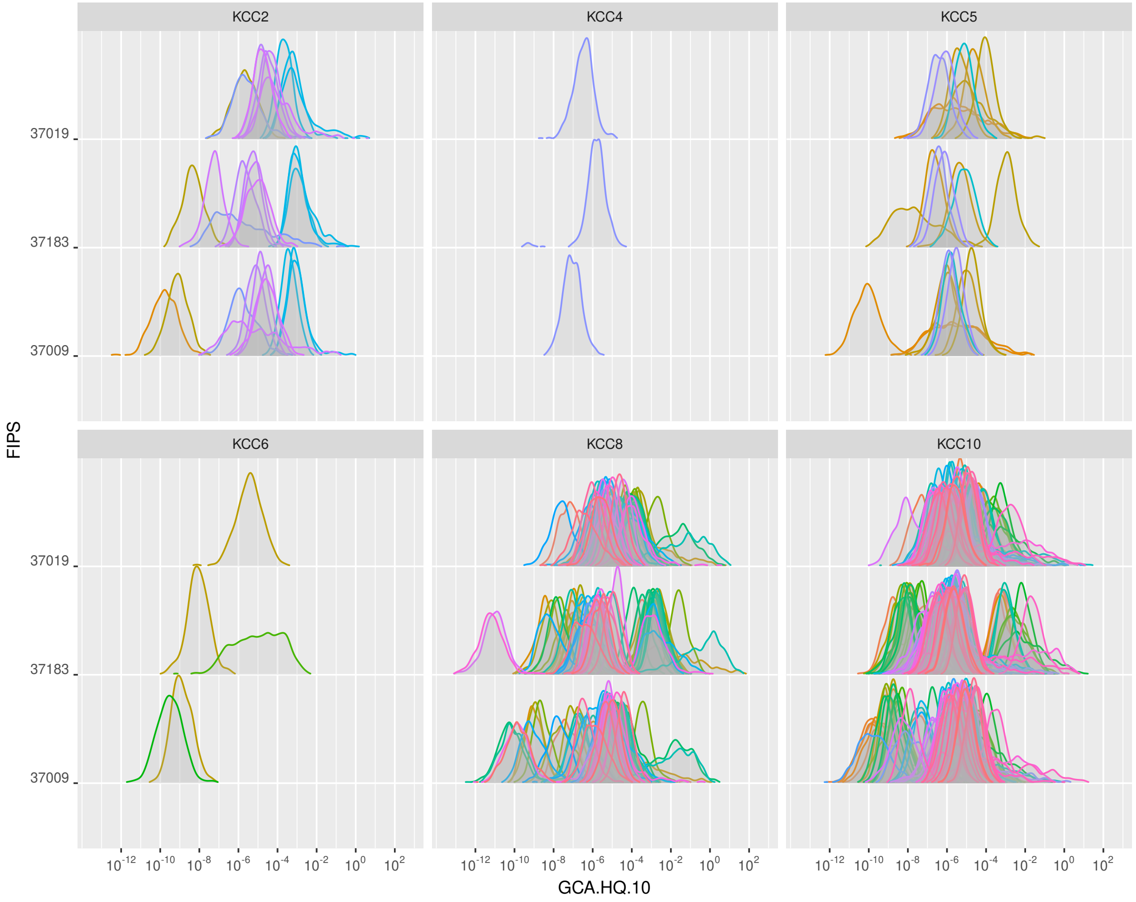

Figure S3: Hazard Quotient density by KCC mode of action
